# Expanding the Epidemiologia e Serviços de Saúde associate editor’s team: strengthening editorial diversity

**DOI:** 10.1590/S2237-96222025v34e20252004.en

**Published:** 2025-12-08

**Authors:** Taís Freire Galvão, Maria Auxiliadora Parreiras Martins, Everton Nunes da Silva, Jorge Otávio Maia Barreto

**Affiliations:** 1 Universidade Estadual de Campinas, Faculdade de Ciências Farmacêuticas, Campinas, SP, Brazil; 2 Universidade Federal de Minas Gerais, Faculdade de Farmácia, Belo Horizonte, MG, Brazil; 3 Universidade de Brasília, Faculdade de Ciências e Tecnologias em Saúde, Brasília, DF, Brazil; 4 Fundação Oswaldo Cruz, Gerência Regional de Brasília, Brasília, DF, Brazil

On September 22, 2025, spring began in the Southern Hemisphere. The start of the equinox coincided with the “5th RESSatona: RESS Peer Review Marathon”, an edition dedicated to candidates for the associate editor of *Epidemiologia e Serviços de Saúde: revista do SUS* (RESS) ^1^.

The selection of new editors began through a public call made in June 2025 ^2^. The increase in manuscript submissions to RESS seen since the improvement of the editorial workflow ^3^, aligned with the commitment to quality and reduction of processing time ^4^, motivated the structuring of a selection process based on an open and transparent procedure that would allow the participation of interested researchers from all over Brazil ^2^. The process represented an innovation to the practice of forming an editorial team, usually based on the relationships and knowledge of the editors who coordinate the journal ^5^.

Eligible candidates underwent online training, in which good peer review practices were presented and discussed, based on a roadmap containing guiding items for the preparation of scientific reviews ^6^ ^,^ ^7^. Once the basic requirements for the critical evaluation of articles were ensured, candidates were asked to issue scientific reviews of manuscripts submitted to RESS. The quality of these reviews was evaluated using the ScholarOne R-score ^8^, the submission system used by the journal. The associate editor responsible for the manuscript evaluated then classified the reviews as “highly relevant” (3.0), “sufficient” (2.0) or “below average” (1.0) ^9^, following criteria standardized by RESS, and structured feedback was sent to the participants for each review. In cases of scores below 2.0, guidance on improving the review was provided individually, with the aim of improving the activity.

Sixty-nine applications were received, of which 39 met the call’s requirements (three articles published in the last five years, PhD in epidemiology or related fields). Ultimately, 22 people participated in the 5th RESSathon in Brasília and began their activities as associate editors, starting in October 2025. The approved candidates issued between six and eight reviews from July to September, with an average R-score of 2.8. The profile of the new editors indicates gender parity and an age range below 40 years. Of the total, 13 work in institutions in the Southeast region, four in the South, three in the Northeast, and two in the Midwest. This result emphasizes the need to plan strategies to attract researchers from the Northern region of Brazil in future selection processes.

The activities of RESS associate editors are voluntary, carried out by researchers with multiple tasks and commitments. Interest in acting as a member of a scientific journal editorial team, despite the absence of financial compensation, reveals the commitment of researchers to evolving academically and collaborating with the dissemination and development of science ^10^. It is known that this task - based on specific experience in editorial work - gives greater quality to the work of these professionals and assists them in their academic careers ^11^. It is important to recognize their dedication, which collectively enables a fundamental step in the production of scientific knowledge.

The incorporation of new associate editors into RESS strengthens the journal’s activities, which focus on scientific quality, diversity and inclusion. While joining the editorial team of a scientific journal fosters the personal and professional development of the researchers, the expansion of expertise within the editorial team broadens the possibilities for scientific evaluation of methods and themes of interest to the Brazilian Unified Health System (*Sistema Único de Saúde*, SUS). As the SUS journal, RESS strengthens its mission to implement inclusive processes that ensure the diversity and improved qualifications of its authors and editors, thereby enabling the dissemination of relevant evidence for the largest universal health system on the planet.
