# Supplementary material for: Expanding the Epidemiologia e Serviços de Saúde associate editor’s team: strengthening editorial diversity
Source: Epidemiol Serv Saude. 2025 Dec 8;34:e20252004. doi: 10.1590/S2237-96222025v34e20252004.en (PMC12696820; doi:10.1590/S2237-96222025v34e20252004.en)
Supplement: Translation

# Translation: Ampliação do corpo de editores associados da *Epidemiologia e Serviços de Saúde*: fortalecimento da diversidade editorial

**DOI:** 10.1590/S2237-96222025v34e20252004.pt

**Authors:** Taís Freire Galvão, Maria Auxiliadora Parreiras Martins, Everton Nunes da Silva, Jorge Otávio Maia Barreto

**Affiliations:** 1 Universidade Estadual de Campinas, Faculdade de Ciências Farmacêuticas, Campinas, SP, Brasil; 2 Universidade Federal de Minas Gerais, Faculdade de Farmácia, Belo Horizonte, MG, Brasil; 3 Universidade de Brasília, Faculdade de Ciências e Tecnologias em Saúde, Brasília, DF, Brasil; 4 Fundação Oswaldo Cruz, Gerência Regional de Brasília, Brasília, DF, Brasil

Em 22 de setembro de 2025, começou a primavera no Hemisfério Sul. O início do equinócio coincidiu com a realização da “5ª RESSatona: maratona de revisão por pares da RESS”, edição dedicada a candidatos a editor associado da *Epidemiologia e Serviços de Saúde: revista do SUS* (RESS) ^1^.

A seleção de novos editores iniciou-se por meio de chamada pública lançada em junho de 2025 ^2^. O aumento nas submissões de manuscritos à RESS observado desde o aprimoramento do fluxo editorial implementado ^3^, alinhado ao compromisso de qualidade e redução do prazo de processamento ^4^, motivaram a estruturação de seleção baseada em procedimento aberto e transparente que oportunizasse a participação de pesquisadores interessados de todo o Brasil ^2^. O processo representou inovação à praxe de formação de corpo editorial, usualmente constituídos a partir das relações e conhecimentos dos editores que coordenam o periódico ^5^.

Os candidatos elegíveis passaram por treinamento on-line, no qual foram apresentadas e discutidas boas práticas em revisão por pares, com base em um roteiro contendo itens norteadores da elaboração de pareceres científicos ^6^ ^,^ ^7^. Assegurados os requisitos básicos para a avaliação crítica de artigos, os candidatos eram solicitados a emitir pareceres científicos de manuscritos submetidos à RESS. A qualidade desses pareceres foi avaliada empregando-se o R-score do ScholarOne ^8^, sistema de submissão adotado pela revista. O editor associado responsável pelo manuscrito avaliado classificava então os pareceres em “altamente relevante” (3,0), “suficiente” (2,0) ou “abaixo da média” (1,0) ^9^, seguindo critérios padronizados pela RESS, e o feedback estruturado era encaminhado aos participantes para cada revisão. Em casos de notas abaixo de 2,0, orientações para aprimorar o parecer foram repassadas individualmente, visando ao aperfeiçoamento da atividade.

Foram recebidas 69 inscrições, das quais 39 atenderam aos requisitos da chamada (três artigos publicados nos últimos cinco anos, doutorado em epidemiologia ou áreas afins). Ao final, 22 pessoas participaram da 5ª RESSatona, em Brasília, e iniciaram suas atividades como editores associados, a partir de outubro de 2025. Os candidatos aprovados emitiram entre seis e oito pareceres, de julho a setembro, com média no R-score de 2,8. O perfil dos novos editores indica paridade de gênero e faixa etária abaixo de 40 anos. Do total, 13 atuam em instituições da região Sudeste, quatro no Sul, três no Nordeste e dois no Centro-Oeste. Esse resultado enfatiza a necessidade de planejar estratégias para captar pesquisadores da região Norte do Brasil, em seleções futuras.

As atividades dos editores associados da RESS são voluntárias, desenvolvidas por pesquisadores com múltiplas tarefas e compromissos. O interesse em atuar como membro de corpo editorial de periódicos científicos, a despeito da ausência de compensações financeiras, revela o compromisso de pesquisadores em evoluir academicamente e colaborar com a divulgação e o desenvolvimento científicos ^10^. Sabe-se que essa tarefa - a partir da experiência particular na atuação editorial - agrega qualidade aos profissionais e os auxilia na carreira acadêmica ^11^. É importante reconhecer sua dedicação, que coletivamente viabiliza etapa fundamental na produção do conhecimento científico.

A incorporação de novos editores associados à RESS fortalece a política e as atividades da revista, com ênfase na qualidade científica, diversidade e inclusão. Se, por um lado, a admissão em corpo editorial de periódico científico favorece o desenvolvimento pessoal e profissional dos pesquisadores, por outro, a ampliação de expertises do corpo editorial expande as possibilidades de apreciação científica de métodos e temáticas de interesse do Sistema Único de Saúde (SUS). Enquanto revista do SUS, a RESS fortalece sua missão de implementar processos inclusivos que assegurem a diversidade e a melhor qualificação dos seus autores e editores, e, com isso, possibilitem a disseminação de evidências relevantes para o maior sistema de saúde universal do planeta.
